# Gastric inflammatory myofibroblastic tumor in adults: a case report and literature review

**DOI:** 10.3389/fonc.2026.1865679

**Published:** 2026-07-29

**Authors:** Zhenxuan Chen, Kebing Chen, Yuting Gu, Tao Peng

**Affiliations:** 1 Department of Hepatobiliary Surgery, The First Affiliated Hospital of Yangtze University, The First People’s Hospital of Jingzhou, Jingzhou, China; 2 The First School of Clinical Medicine, Yangtze University, Jingzhou, China; 3 Department of Pathology, The First Affiliated Hospital of Yangtze University, The First People’s Hospital of Jingzhou, Jingzhou, China

**Keywords:** adult, anaplastic lymphoma kinase, gastrointestinal stromal tumor, inflammatory myofibroblastic tumor, stomach neoplasms

## Abstract

Gastric inflammatory myofibroblastic tumor (IMT) is a rare mesenchymal neoplasm of intermediate biological potential, with adult cases being particularly uncommon. We report a 64-year-old man with an incidentally detected gastric mass that was preoperatively suspected to be a gastrointestinal stromal tumor (GIST). The relevant literature on adult gastric IMT was also reviewed. Complete surgical resection with negative margins (R0 resection) remains the mainstay of treatment, whereas patients with confirmed ALK rearrangements may benefit from ALK inhibitor therapy. Histopathological confirmation and long-term surveillance are essential because of the potential for recurrence and occasional aggressive behavior.

## Case presentation

**Figure 1:**
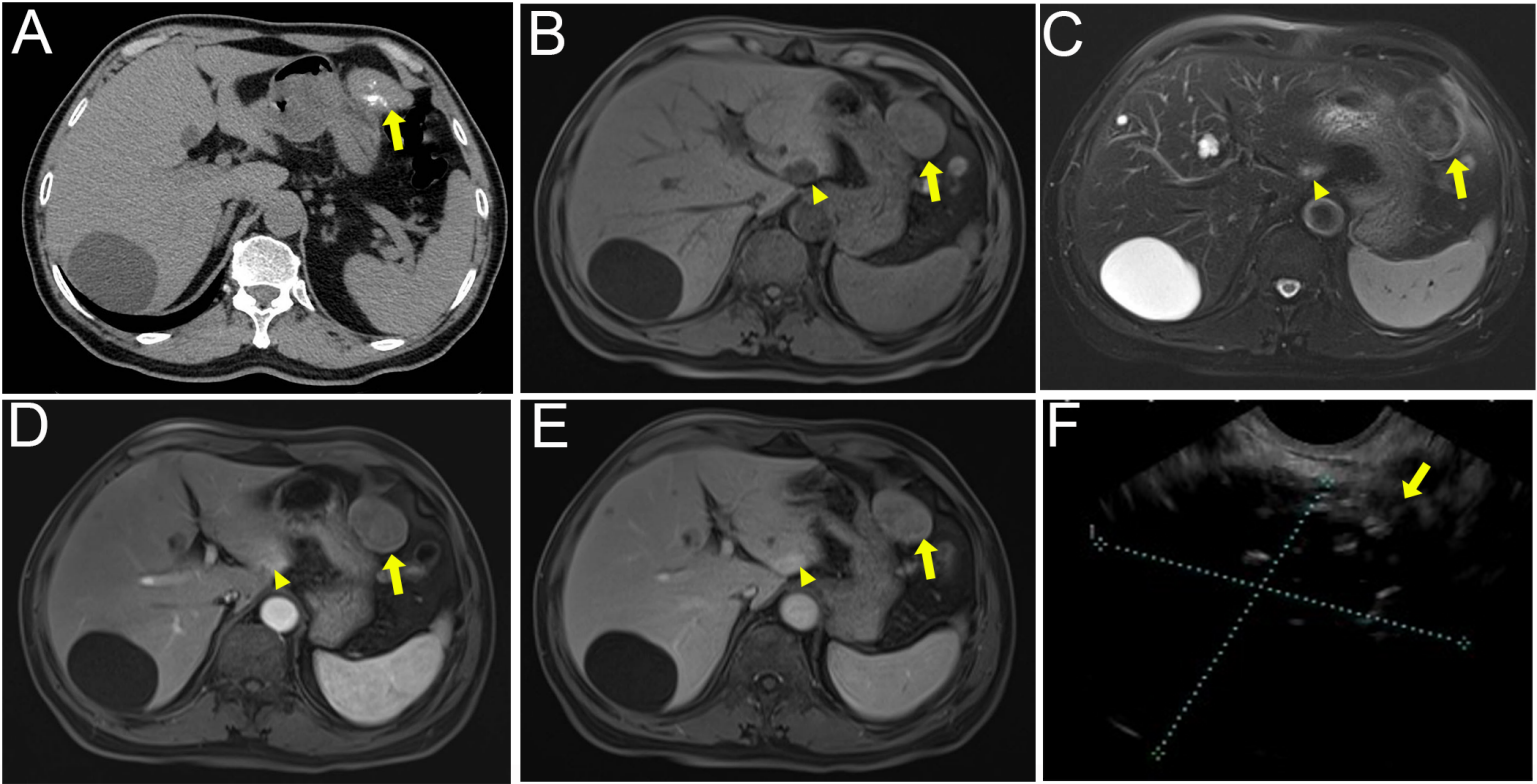
Imaging findings of the gastric IMT and hepatic hemangioma. **(A)** CT showing a heterogeneous mixed-density mass with focal calcifications. **(B, C)** T1- and T2-weighted MRI showing low signal intensity of the gastric mass. **(D, E)** Arterial- and venous-phase contrast-enhanced MRI showing moderate enhancement of the mass. **(F)** EUS showing a well-demarcated hypoechoic-to-anechoic lesion arising from the muscularis propria with an exophytic growth pattern. Arrows indicate the GIMT, and arrowheads indicate the hepatic hemangioma.

A 64-year-old man presented to our hospital after a liver lesion was incidentally detected on abdominal ultrasonography during a routine health examination at another hospital. The patient was asymptomatic, and physical examination on admission revealed no significant abnormalities. Laboratory tests showed unremarkable liver function and coagulation profiles. Serum tumor markers, including carbohydrate antigen 19-9 (CA19-9), alpha-fetoprotein (AFP), carcinoembryonic antigen (CEA), and carbohydrate antigen 125 (CA125) were within the normal range. During further evaluation after admission, a gastric mass was identified. Computed tomography (CT) showed a heterogeneous mixed-density mass measuring approximately 40 mm× 42 mm in the left upper abdomen with focal calcifications. No obvious necrotic areas, enlarged regional lymph nodes, or ascites were observed (**Figure 1A**). Enhanced magnetic resonance imaging (MRI) confirmed that the liver lesion was consistent with a hemangioma. The gastric lesion was oval and showed low signal intensity on both T1-weighted and T2-weighted images. It was closely related to the gastric wall and clearly separated from the adjacent intestinal structures (**Figures 1B, C**). The lesion demonstrated moderate enhancement during both the arterial and venous phases (**Figures 1D, E**). Endoscopic ultrasonography (EUS) revealed a well-demarcated hypoechoic-to-anechoic lesion located in the proximal stomach along the greater curvature. The lesion originated from the muscularis propria of the gastric wall and exhibited an exophytic growth pattern, measuring approximately 45 mm×37 mm. Internal heterogeneous echogenicity with focal calcifications was observed. No obvious internal vascularity or cystic degeneration was detected on EUS. The surrounding gastric wall layers were preserved, and no enlarged regional lymph nodes were identified (**Figure 1F**). Based on the exophytic growth pattern and apparent origin from the muscularis propria on imaging, a gastrointestinal stromal tumor (GIST) was considered the most likely preoperative diagnosis.

**Figure 2:**
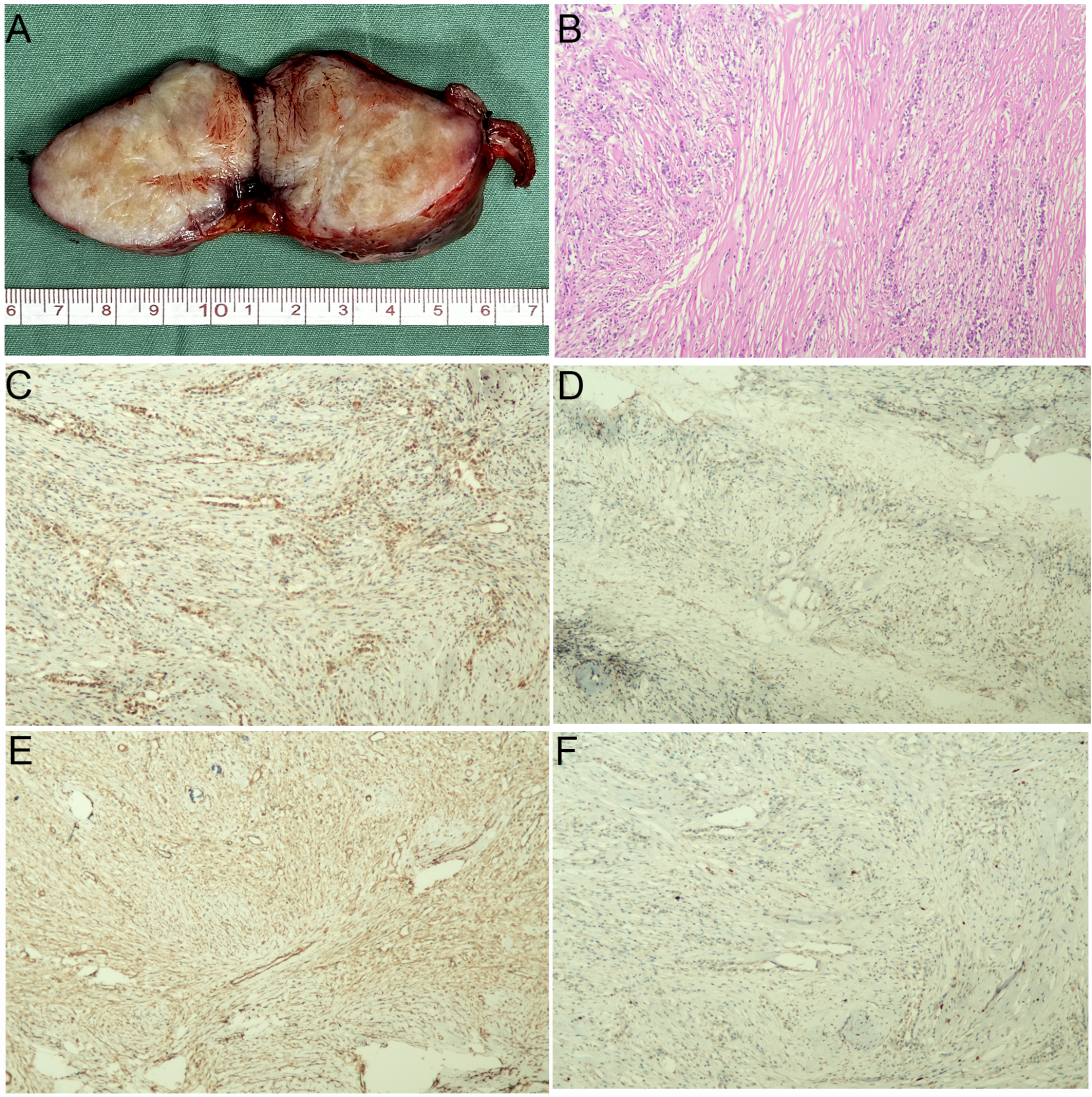
Gross and histopathological findings of the gastric IMT. **(A)** Gross appearance showing a firm mass with a grayish-white cut surface. **(B)** Spindle cell proliferation with a prominent inflammatory infiltrate (H&E staining). **(C)** Focal weak ALK immunoreactivity with the 1A4 clone. **(D)** Negative ALK staining with the D5F3 clone. **(E)** Weak SMA positivity. **(F)** Low Ki-67 labeling index of approximately 2%. Original magnification for **(B–F)**, ×200.

The patient underwent laparoscopic wedge resection, and the mass was successfully excised en bloc. Gross examination revealed a firm mass with a grayish-white cut surface (**Figure 2A**). Histopathological examination of hematoxylin and eosin (H&E)-stained sections showed a spindle cell proliferation accompanied by a prominent inflammatory infiltrate composed predominantly of plasma cells and lymphocytes, with focal calcifications (**Figure 2B**). No tumor necrosis was identified. Immunohistochemically, the tumor cells showed focal weak ALK immunoreactivity with the 1A4 clone (**Figure 2C**), whereas staining with the D5F3 clone was negative (**Figure 2D**). The tumor cells were weakly positive for smooth muscle actin (SMA) (**Figure 2E**) and focally positive for desmin, pan-cytokeratin, and epithelial membrane antigen (EMA). DOG1, CD117, S100, SOX10, and β-catenin were negative. The Ki-67 labeling index was approximately 2% (**Figure 2F**). Together with the characteristic histomorphological findings, this immunophenotype supported a diagnosis of inflammatory myofibroblastic tumor (IMT) and argued against GIST. The absence of S100 and SOX10 expression made gastrointestinal neuroectodermal tumor (GNET) unlikely, while the lack of marked cytological atypia and tumor necrosis, the low proliferative index, and only weak or focal expression of smooth-muscle markers did not support leiomyosarcoma. The patient was followed up for 2 years after surgery. At 6 months postoperatively, abdominal CT revealed no evidence of recurrence or metastasis. Thereafter, telephone follow-up was conducted every 6 months. At the 2-year follow-up, the patient remained clinically well and reported no symptoms suggestive of recurrence.

## Literature review methodology

PubMed was searched from database inception through March 31, 2026, using combinations of the following terms: (“gastric” OR “stomach”) AND (“inflammatory myofibroblastic tumor” OR “inflammatory myofibroblastic tumour” OR “inflammatory pseudotumor”). The reference lists of relevant articles were manually screened to identify additional eligible reports. Adult patients, defined as those aged 18 years or older, with histologically confirmed gastric inflammatory myofibroblastic tumor (IMT) were included. Tumors arising outside the stomach, duplicate publications, and reports lacking sufficient clinicopathological information were excluded. When multiple publications described the same patient, the report containing the most complete clinical-pathological and follow-up information was retained. Data on patient demographics, clinical presentation, tumor location and size, histopathological and immunohistochemical findings, treatment, and follow-up were extracted. No language restrictions were applied.

## Discussion

### Findings from the narrative review

A total of 47 previously reported adult patients with gastric inflammatory myofibroblastic tumor (IMT) met the inclusion criteria. The present case was not included in the literature-derived cohort or in the summary analyses. The individual characteristics of the included patients are presented in **Table 1**, and their overall characteristics are summarized in **Table 2**.

**Table 1:** Individual characteristics of 47 previously reported adult gastric IMT cases.

NO.	Author / year	Age / Sex	Presenting symptoms	Tumor location in the stomach	Size (cm)	ALK status	Treatment	Local invasion / metastasis	Follow-up	
									Duration (months)	Status
1	Wei et al.,2015 (23)	19 /M	Melena, dizziness, blackout, fatigue, hematemesis, upper abdominal discomfort, severe anemia	Gastric body, greater curvature, near the fundus	5.2	Negative	Gastric tumor resection	NR	2	NED
2	Chukkalore et al., 202 (44)	42 / F	Melena, generalized weakness, severe acute blood-loss anemia	Gastric fundus extending into the body, posterior wall	5.4	Negative	Robotic partial gastrectomy with intraoperative EGD	NR	6	NED
3	Desai et al., 2023 (24)	36/M	Dyspnea, severe abdominal distension, abdominal pain, early satiety, weight loss, low-grade fever	Lesser sac; arising from the gastric submucosa	34	Negative	En bloc tumor resection with wedge gastrectomy and wedge hepatectomy	Liver	6	NED
4	Chen et al., 2015 (25)	50 / F	Left-sided abdominal distension, palpable abdominal mass, anemia	Between the greater curvature of the stomach and spleen	22	Negative	Total gastrectomy + splenectomy	Spleen	4	NED
5	Fan et al., 2017 (26)	37 / M	Upper abdominal pain	Gastric angular incisure (lesser curvature)	4.5	Positive	Total gastrectomy + lymph-node (LN) dissection	Regional LN metastasis (5/19)	6	Asymptomatic at 6 months; recurrence status NR
6	Song et al., 2021 (27)	43 / M	Upper abdominal pain + melena	Gastric antrum	4.5	NR	Distal subtotal gastrectomy	NR	70	NED
7	Wang et al., 2021 (28)	27 / M	NR	Gastric antrum	3.2	Focally positive	Surgical resection	NR	38	NED
8		41 / M	NR	Gastric body	6.1	Focally positive	Surgical resection	NR	12	NED
9		22 / F	NR	Gastric body	6.8	Focally positive	Surgical resection	NR	34	NED
10	Lee et al., 2018 (29)	43 / F	Asymptomatic	Gastric body	3.4	NR	Wedge resection	None	132	NED
11		36 / F	Melena	Gastric Antrum	3.0	NR	Wedge resection	None	72	NED
12		38 / F	Epigastric pain	Gastric Cardia	4.2	NR	Wedge resection	None	48	NED
13		39 / F	Dyspepsia	Gastric Cardia	2.0	NR	Wedge resection	None	36	NED
14		35 / F	Asymptomatic	Gastric body	2.5	NR	Wedge resection	None	36	NED
15	He et al., 2018 (30)	68 / F	Dysphagia	Gastric cardia, posterior wall	4.5	Negative	Complete tumor resection + splenectomy + partial diaphragm resection	Spleen + diaphragm	8	NED
16	Park et al., 2008 (31)	55 / F	Acute sharp abdominal pain, anemia	Gastric body, anterior wall along the greater curvature; exophytic	8.5	Negative	Open gastric wedge resection with greater omentum	None	NR	NED
17	Mohammad Hoseini-Azar et al., 2016 (32)	18 / M	Nausea, epigastric pain, intermittent fever, night sweats, weight loss, early satiety, weakness, prior hematemesis, anemia	Gastric fundus extending to the greater curvature	10	Negative	Laparoscopic tumor resection + partial gastrectomy + splenectomy	Spleen	9	NED
18	Huang et al., 2024 (33)	57 / M	Epigastric pain, melena, poor appetite, malaise, severe anemia	Gastric body–antrum junction along the lesser curvature	4	Negative	Laparoscopic subtotal gastrectomy with Billroth II and Braun reconstruction; subsequent incomplete cardiac tumor debulking; doxorubicin	Cardiac involvement, likely metastatic	5.5	DOD
19	Ning et al., 2017 (34)	50 / F	Intermittent epigastric pain	Gastric antrum	3	NR	Endoscopic submucosal dissection (ESD)	NR	24	NED
20	Zou et al., 2025 (35)	30 / F	Melena, nausea, vomiting	Posterior wall of the gastric fundus extending to the cardia	4	Positive	Total gastrectomy (D2) + splenectomy + Roux-en-Y reconstruction	Esophagus and spleen	36	NED
21	Fong et al., 2010 (36)	56 / M	Epigastric pain	Gastric body (posterior wall; exophytic)	10	NR	Surgical resection	NR	NR	NR
22	Hiramatsu et al., 2024 (37)	69 / M	Fatigue, weakness, severe anemia, fever	Gastric body (lesser curvature; intraluminal)	8	Negative	Distal gastrectomy + cholecystectomy (Roux-en-Y)	None	18	NED
23	Hayashi et al., 2018 (38)	53 / F	Asymptomatic	Gastric lesser curvature (lower third)	2	Positive	Combined laparoscopic and endoscopic wedge resection	NR	9	NED
24	Fernandez Rodriguez et al., 2023 (39)	45 / F	Diffuse abdominal pain	Gastric fundus/body (anterior surface; perigastric)	1.3	Negative	Laparoscopic wedge resection	NR	1	Alive; recurrence status NR
25	Hattori et al., 2024 (40)	69 / M	Asymptomatic (incidental finding)	Posterior wall of the middle third of the stomach, near the greater curvature	3.5	Negative	Laparoscopic–endoscopic cooperative wedge resection (LECS)	NR	29	NED
26	Hošala et al. / 2025 (41)	24 / M	Anemia; no abdominal pain, weight loss, or GI bleeding	Anterior wall of the middle third of the stomach	6.5	Positive	Laparoscopic gastric wedge resection	NR	30	NED
27	Qiu et al. / 2014 (42)	61 / F	High fever (39.8 °C), intermittent epigastric pain, abdominal distention, anemia	Lesser curvature near angular incisure (gastric antrum region)	3	Negative	Distal gastrectomy with D2 lymph node dissection (Billroth I reconstruction)	None	3	NED
28	Albayrak et al. / 2010 (43)	56 / F	Hematemesis, melena, intermittent nausea and vomiting (5 months), anemia	Cardia (extending toward pylorus; gastric outlet obstruction)	11	Negative	Partial gastrectomy (Billroth I)	None	8	No recurrence reported; symptom-free
29	Kim et al. / 2015 (44)	28 / M	Syncope, hematemesis, severe anemia	Proximal stomach (fundus, cardia, upper body; nearly entire proximal stomach)	4	ALK rearrangement negative; IHC NR	Laparoscopic proximal gastrectomy with double-tract anastomosis	NR	18	NED
30	Arslan et al. / 2013 (45)	65 / F	Dyspepsia, intermittent epigastric pain, loss of appetite, nausea, vomiting, and weight loss	Antrum (submucosal gastric lesion with fistula)	11	Positive	Surgical wedge resection	NR	37	NED
31	Katakwar et al. / 2014 (46)	45 / M	Weight loss, epigastric pain (1 year), anemia	Pylorus / lower body (posterior wall, near lesser curvature)	5.7	Positive	Distal gastrectomy + Roux-en-Y; reoperation (bypass) due to recurrence	None	1	Recurrence reported at 1 month; alive after reoperation, final disease status NR
32	Shi et al. / 2010 (47)	36 / M	Abdominal pain, abdominal mass	Antrum (lesser curvature)	4.5	Positive	Partial gastrectomy	NR	60	NED
33		42 / M	Abdominal pain, mass, GI bleeding	Upper body (greater curvature)	8.0	Positive	Partial gastrectomy + reoperation	NR	24	Recurrence at 12 months; NED 11 months after second surgery
34		40 / M	Abdominal mass	Upper body	6.3	Positive	Partial gastrectomy	NR	40	NED
35		45 / M	Abdominal pain, mass	Gastric angle	5.5	Positive	Partial gastrectomy	NR	31	NED
36		40 / F	Abdominal pain, mass	Lower body (posterior wall)	5.8	Positive	Partial gastrectomy	NR	48	NED
37	Flores-Trujillo et al. / 2022 (48)	52 / F	Epigastric pain, intolerance to solids and liquids, nausea, porraceous vomiting, melena, gastric outlet obstruction	Distal body / pyloric antrum, posterior surface, near lesser curvature;	10	Negative	D1 subtotal gastrectomy + Billroth II with Braun enteroenterostomy	Duodenum	NR	NED
38	Hajong et al. / 2021 (49)	25 / F	Upper abdominal pain, palpable swelling	Body + antrum (anterior wall, exophytic)	10	NR	Distal gastrectomy + Roux-en-Y	None	6	No recurrence reported
39	Ribeiro et al. / 2012 (50)	37 / F	Epigastric pain, weight loss, anemia	Posterior wall of gastric body	9	Positive	Subtotal gastrectomy + Billroth I	NR	NR	NED
40	Laamiri et al. / 2024 (51)	55 / F	Abdominal pain, weight loss, anemia	Posterior wall of gastric body (near lesser curvature)	2	Negative	Wedge resection (wide local excision)	NR	6	NED
41	Masuda et al., 2024 (52)	39/M	Asymptomatic, incidental finding	Gastric fornix, greater curvature/anterior wall	2.5	Negative	Local resection using NEWS	NR	48	NED
42	Cheng et al., 2018 (53)	52/F	Upper abdominal pain, acid reflux and belching for 2 months	Gastric antrum, exophytic	4.3	Positive	Laparoscopic-assisted distal gastrectomy	None	6	NED
43	Jain et al., 2012 (54)	35/F	Abdominal pain, fever, vomiting, loss of appetite, anemia	Greater curvature; transmural with intraluminal and exophytic components	11	Negative	Wide local excision with a 1.5-cm rim of normal gastric wall	None	7	Alive and well at 7 months; recurrence status NR
44	Jadhav et al., 2019 (55)	18/M	Epigastric mass, loss of appetite, weight loss, anemia	Lesser curvature, just below the gastroesophageal junction; exophytic	9	Negative	Local tumor excision	None	60	NED
45	Kim et al., 2004 (56)	26/M	Enlarging palpable left abdominal mass, anemia	Distal esophagus to gastric fundus/high body, predominantly posterior wall	8	NR	Total gastrectomy + distal pancreatectomy + splenectomy + transverse colectomy + lymph-node dissection	Pancreas, spleen, multiple regional lymph nodes	1.2	Peritoneal dissemination at 5 weeks after surgery
46	Leon et al., 2006 (57)	50/F	Persistent vomiting, early satiety, and weight loss	Posterior wall of the gastric remnant, near the gastrojejunostomy	7	NR	Local tumor resection followed by subtotal gastrectomy with Billroth II reconstruction	None	24	NED
47	Bjelovic et al., 2013 (58)	43/F	Epigastric pain, nausea, and pyrosis	Distal stomach, posterior wall along the lesser curvature, below the angular incisure	6	Positive	Subtotal gastrectomy + D2 lymph-node dissection + Roux-en-Y reconstruction	None	24	NED

M, male; F, female; ALK, anaplastic lymphoma kinase; LN, lymph node; EGD, esophagogastroduodenoscopy; ESD, endoscopic submucosal dissection; LECS, laparoscopic–endoscopic cooperative surgery; NEWS, non-exposed endoscopic wall-inversion surgery; NED, no evidence of disease; DOD, died of disease; NR, not reported. Adults were defined as patients aged 18 years or older. Tumor size was preferentially based on the resected pathological specimen. When pathological size was unavailable or did not represent the entire lesion, the largest radiological, endoscopic, or intraoperative diameter was used. ALK status was recorded according to the testing method reported in each study; immunohistochemistry and molecular testing were not uniformly performed. None indicates that the absence of invasion or metastasis was explicitly reported; NR indicates that the relevant information was not reported. Gastric-remnant IMT after previous gastrectomy was included and specifically identified in the table.

**Table 2:** Summary of 47 previously reported adult gastric IMT cases.

Variable	Value
Age, years	42.38 ± 13.46
Sex, n (%)	
Male	21 (44.7%)
Female	26 (55.3%)
Tumor size, cm	5.50 (3.75–8.25)
ALK status, n/N (%)	
Negative	19/36 (52.8%)
Positive	17/36 (47.2%)
Missing, n (%)	11 (23.4%)
Tumor location, n (%)	
Proximal stomach	13 (27.7%)
Gastric body	13 (27.7%)
Distal stomach	14 (29.8%)
Other/multiregional	7 (14.9%)
Common symptoms, n (%)	
Abdominal pain	24 (51.1%)
Anemia	17 (36.2%)
GI bleeding/melena	11 (23.4%)
Abdominal mass	9 (19.1%)
Weight loss	8 (17.0%)
Nausea/vomiting	8 (17.0%)
Fever	5 (10.6%)
Asymptomatic/incidental finding	5 (10.6%)
Treatment, n (%)	
Local resection	21 (44.7%)
Gastrectomy	16 (34.0%)
Complex treatment	10 (21.3%)
Local invasion/metastasis, n (%)	
Present	9 (19.1%)
Explicitly absent	16 (34.0%)
Not reported	22 (46.8%)
Follow-up duration, months	24.0 (6.0–36.5), n = 43
Aggressive clinical behavior, n (%)	
Local invasion or metastatic involvement	9 (19.1%)
Direct invasion of adjacent organs	7 (14.9%)
Regional lymph-node metastasis	2 (4.3%)
Distant or disseminated disease	2 (4.3%)
Local recurrence during follow-up	2 (4.3%)
Explicitly absent	16 (34.0%)
Not reported	22 (46.8%)

Data are presented as mean ± standard deviation or median (interquartile range), as appropriate; ALK percentages for positive and negative tumors were calculated using the 36 cases with reported ALK results; missing ALK data were calculated using all 47 cases; Symptoms were non-mutually exclusive; therefore, percentages do not sum to 100%; Proximal stomach included the cardia, fundus/fornix, gastroesophageal-junction region, and upper gastric body. The distal stomach includes the lower third, the angle/angular incisure, the antrum, and the pylorus. Multiregional, diffuse, gastric-remnant, lesser-sac, or otherwise unassignable tumors were classified as other; local resection included local tumor excision, wedge resection, ESD, LECS/NEWS, and unspecified surgical resection without explicit gastrectomy. Gastrectomy included partial, subtotal, distal, proximal, or total gastrectomy without multivisceral resection, reoperation, or systemic therapy. Complex treatment included multivisceral resection, reoperation, or systemic therapy; follow-up duration was available for 43 patients. Absent indicates that the original report explicitly documented no local invasion or metastasis; not reported indicates that the relevant information was unavailable and was not considered evidence of absence.

The mean age of the patients was 42.38 ± 13.46 years, and 26 patients (55.3%) were female. The median tumor size was 5.50 cm (interquartile range, 3.75–8.25 cm). Abdominal pain was the most common presenting symptom, occurring in 24 patients (51.1%), followed by anemia in 17 (36.2%) and gastrointestinal bleeding or melena in 11 (23.4%). ALK status was reported in 36 patients, of whom 17 (47.2%) were ALK-positive. Local resection was performed in 21 patients (44.7%), gastrectomy in 16 (34.0%), and complex treatment in 10 (21.3%). Follow-up information was available for 43 patients, with a median follow-up duration of 24.0 months.

Direct invasion of adjacent organs was reported in seven patients (14.9%). Definite metastatic disease was documented in two patients (4.3%), and probable cardiac metastasis was described in one additional patient. Regional lymph-node metastases were reported in two patients, whereas postoperative peritoneal dissemination occurred in one. Local recurrence was documented in two patients. However, information regarding invasion or metastasis was not explicitly reported in 22 cases. Therefore, these proportions are descriptive and should not be interpreted as estimates of the true incidence of metastatic gastric IMT.

### WHO classification and diagnostic implications

According to the current World Health Organization Classification of Tumours of the Digestive System, IMT is classified among mesenchymal tumors of the digestive system, within the group of adipose tissue and (myo) fibroblastic tumors, rather than among epithelial gastric malignancies or gastric carcinomas (1). Gastric IMT is therefore regarded as a mesenchymal neoplasm of intermediate biological potential.

Gastric IMT may clinically and radiologically mimic other gastric mesenchymal tumors, particularly GIST, GNET, and leiomyosarcoma. GIST is the most important differential diagnosis because both tumors may arise from the muscularis propria and exhibit an exophytic growth pattern (2–4). Histologically, GIST is composed of spindle or epithelioid cells, generally without the prominent plasma cell- and lymphocyte-rich inflammatory background characteristic of IMT. Most GISTs express DOG1 and CD117, whereas IMTs are generally negative for these markers. In our literature-derived cohort, 17 of 36 patients with available ALK data (47.2%) were reported as ALK-positive. Previous studies have also demonstrated variable expression of SMA, desmin, and cytokeratins in IMT (5). Nevertheless, negative DOG1 and CD117 staining alone does not completely exclude GIST, and the distinction should be based on the combined morphological, immunohistochemical, and, when necessary, molecular findings.

GNET should also be considered in the differential diagnosis of gastric mesenchymal tumors (6–8). It is typically composed of relatively uniform epithelioid or spindle cells and shows diffuse expression of S100 protein and SOX10, often in association with an EWSR1 rearrangement, whereas melanocytic markers are generally negative. In contrast, IMT usually displays a prominent inflammatory infiltrate and lacks diffuse S100 and SOX10 expression.

Leiomyosarcoma is characterized by intersecting fascicles of spindle cells with eosinophilic cytoplasm, cytological atypia, increased mitotic activity, and, occasionally, tumor necrosis (9–11). It typically shows diffuse expression of smooth-muscle markers, including SMA, desmin, and h-caldesmon, while DOG1 and CD117 are negative. Because SMA expression may also occur in IMT, SMA positivity alone is insufficient to establish smooth-muscle differentiation. The intensity and distribution of SMA and desmin expression should therefore be interpreted together with h-caldesmon staining and the histomorphological findings.

In the present case, spindle-cell proliferation accompanied by prominent plasma cell and lymphocyte infiltration strongly favored IMT. DOG1 and CD117 negativity made GIST less likely, while the absence of S100 and SOX10 expression argued against GNET. The absence of tumor necrosis, the low Ki-67 labeling index, and only weak or focal expression of SMA and desmin made leiomyosarcoma less likely. ALK immunoreactivity was focally and weakly positive with the 1A4 clone but negative with the D5F3 clone. Although this discordant staining pattern was not definitive evidence of an ALK rearrangement, the overall histomorphological and immunohistochemical findings supported the diagnosis of gastric IMT.

### Treatment and ALK-targeted therapy

Complete surgical resection with negative margins remains the preferred treatment for localized and resectable gastric IMT. For unresectable, recurrent, refractory, or metastatic ALK-positive IMT, ALK-targeted therapy represents an important therapeutic option (12). Crizotinib is the most extensively studied ALK inhibitor in IMT and has demonstrated durable activity in patients with advanced or inoperable ALK-positive tumors (13). It has been approved by the U.S. Food and Drug Administration for adult and pediatric patients aged 1 year or older with unresectable, recurrent, or refractory ALK-positive IMT (14, 15).

Other ALK inhibitors, including ceritinib, alectinib, brigatinib, and lorlatinib, have also been used in selected patients, particularly after resistance or intolerance to crizotinib (12, 13). However, the evidence supporting these later-generation agents in IMT is currently based mainly on case reports, small series, and limited clinical experience. In the present case, ALK staining was focally and weakly positive, although molecular testing for an ALK rearrangement was not performed. Therefore, the immunohistochemical result should not be interpreted as definitive evidence of an ALK rearrangement, and molecular confirmation would be required before considering ALK-targeted therapy.

### Metastatic potential and prognosis

IMT is considered a mesenchymal neoplasm of intermediate biological potential. Although distant metastasis is uncommon, it has been reported in fewer than 5% of IMTs arising at different anatomical sites, with the lung being the most frequently involved metastatic site; the brain, liver, and bone may also be affected (14, 16). These figures are derived from IMTs arising at various sites and should not be interpreted as the specific metastatic rate of gastric IMTs.

Margin-negative resection is the most consistently recognized favorable prognostic factor, whereas incomplete resection is associated with an increased risk of local recurrence (17, 18). Histopathological features associated with aggressive behavior include tumor necrosis, lymphovascular invasion, increased mitotic activity, high cellularity, and infiltrative margins (19). The prognostic significance of ALK expression remains uncertain, as published studies have yielded inconsistent results regarding its association with recurrence and metastasis (20, 21). Of particular note, epithelioid inflammatory myofibroblastic sarcoma (EIMS) is a rare and highly aggressive variant of IMT that is frequently associated with RANBP2–ALK fusion and follows a distinctly unfavorable clinical course (22).

## Data Availability

The original contributions presented in the study are included in the article/supplementary material. Further inquiries can be directed to the corresponding author.
